# Quality of Life of Colorectal Cancer Survivors: Mapping the Key Indicators by Expert Consensus and Measures for Their Assessment

**DOI:** 10.3390/healthcare12121235

**Published:** 2024-06-20

**Authors:** Urška Smrke, Sara Abalde-Cela, Catherine Loly, Jean-Paul Calbimonte, Liliana R. Pires, Simon Lin, Alberto Sánchez, Sara Tement, Izidor Mlakar

**Affiliations:** 1 Faculty of Electrical Engineering and Computer Science, University of Maribor, 2000 Maribor, Slovenia; 2 RUBYnanomed LDA, Praça Conde de Agrolongo, 4700-314 Braga, Portugal; 3 Gastroenterology Department, University Hospital of Liège, 4000 Liège, Belgium; 4 Institute of Informatics, University of Applied Sciences and Arts Western Switzerland HES-SO, 3960 Sierre, Switzerland; 5 The Sense Innovation & Research Center, 1007 Lausanne, Switzerland; 6 Science Department, Symptoma GmbH, 5020 Vienna, Austria; 7 Department of Internal Medicine, Paracelsus Medical University, 5020 Salzburg, Austria; 8 Department of eHealth, Galician Research & Development Center in Advanced Telecommunications (GRADIANT), 26334 Vigo, Spain; 9 Department of Psychology, Faculty of Arts, University of Maribor, 2000 Maribor, Slovenia

**Keywords:** quality of life, surveys and questionnaires, adult oncology, colorectal cancer survivors, Delphi study, scoping review, expert consensus

## Abstract

Quality of life (QoL) assessments are integral to cancer care, yet their effectiveness in providing essential information for supporting survivors varies. This study aimed to elucidate key indicators of QoL among colorectal cancer survivors from the perspective of healthcare professionals, and to evaluate existing QoL questionnaires in relation to these indicators. Two studies were conducted: a Delphi study to identify key QoL indicators and a scoping review of questionnaires suitable for colorectal cancer survivors. Fifty-four healthcare professionals participated in the Delphi study’s first round, with 25 in the second. The study identified two primary QoL domains (physical and psychological) and 17 subdomains deemed most critical. Additionally, a review of 12 questionnaires revealed two instruments assessing the most important general domains. The findings underscored a misalignment between existing assessment tools and healthcare professionals’ clinical priorities in working with colorectal cancer survivors. To enhance support for survivors’ QoL, efforts are needed to develop instruments that better align with the demands of routine QoL assessment in clinical practice.

## 1. Introduction

The burden of cancer incidence and mortality is rapidly growing, reflecting population growth and ageing, among other factors [1]. It is estimated that the cancer burden will increase globally by almost 50% from 2020 to 2040 [1,2]. That is, while cancer death rates decreased significantly in the last few decades (e.g., in the United States by 33% from 1991 to 2020), survival rates are increasing (e.g., for all types of cancer combined the five-year relative survival rate increased from 49% in the mid-1970s to 68% in the 2010s; [3]), which holds true also for generally more lethal cancers [4]. One of the most burdensome cancers is colorectal cancer, being the second leading cause of cancer-related death [1] and the third most common cancer in males and females [1,5], representing 10.7% of new cases in 2020 [2], with its incidence still expected to grow [6]. With the current five-year survival rates in most developed countries ranging from 50% to over 70% [4,6,7] and increasing, the population of colorectal cancer survivors is also growing [8].

Cancer survivorship is being recognized as an essential component of cancer care [9]. However, cancer survivors, i.e., those who have completed primary cancer treatment [7], often start to suffer from physical disability, distress, and reduced quality of life (QoL) over time [10,11]. The burden of cancer and cancer treatment is therefore not evident only in terms of morbidity and mortality, but also in the impact on survivors’ QoL in the long term [12]. Overall burden of cancer diagnosis, cancer symptomatology and other clinical characteristics (e.g., fatigue, difficulty sleeping, psychological challenges), and intensive treatment pattern (e.g., radiotherapy, chemotherapy, colostomy [5,13]) have been found to negatively impact various aspects of survivors’ QoL. As many as 30–40% of cancer survivors report at least mild levels of anxiety and depression [14], which may interfere with the ability to effectively cope with cancer diagnosis, symptoms, and treatment. Additionally, cancer survivors also suffer from negative impacts on other areas of life directly linked to cancer, such as treatment adherence and healthcare utilization [15], but also importantly on various areas of QoL [12], e.g., physical, psychological, and social QoL. A recent meta-analysis [5] found that colorectal cancer survivors have a 51% increased risk of experiencing depression after diagnosis and do experience high levels of anxiety and depression regarding health-related QoL and mortality. Even though the major deterioration of health-related QoL occurs during the first six months after diagnosis and treatment [8], many cancer survivors still report negatively impacted QoL well into the survivorship period [8,15]. Improving the QoL of colorectal cancer survivors is important, not only from the survivors’ perspective of wanting to be provided with appropriate and tailored care, but also as the growing population of survivors poses an increasing burden on healthcare systems due to their persistent health problems and decreased levels of QoL [8,15]. This need is also reflected in the actions promoted by the European Commission, such as Mission Cancer, recognizing QoL as one of the most important areas of intervention [16], and the EU Beating Cancer Plan, urging a change of focus from the length of life after diagnosis to length and QoL after diagnosis [17].

With increasing attention on addressing survivors’ QoL in research and clinical care (e.g., [18], important improvements have been made in focusing on what matters most to patients, together with providing insights into the importance of the aspects relevant to healthcare professionals (HCPs), even though these do not always completely align [12,19]. Of course, to improve QoL, it first needs to be measured. Routine assessment of QoL has been shown to have a role in improving outcomes, such as overall survival, functional outcomes, and health-related QoL [12]. Additionally, such assessments have been repeatedly recommended as an important part of cancer care, meeting the patients’ needs to a broader extent than physical issues. This also contributes to ensuring that appropriate further care and support is provided to the survivors [20].

Even though historically QoL has not been addressed and assessed as rigorously as traditional disease-related outcomes (e.g., overall survival), important improvements have been achieved, especially in the last decade [12]. A plethora of questionnaires was developed for the assessment of the QoL of cancer patients and survivors. These questionnaires differ in their intended populations: from those for the general population but used in the colorectal cancer population (e.g., SF-36v2 Health survey (SF-36v2), [21]; World Health Organization Quality-of-Life Scale (WHOQOL-BREF) [22]) and the cancer survivors population (e.g., Quality of Working Life Questionnaire for Cancer Survivors (QWLQ-CS), [23]) to those specific to colorectal cancer (e.g., Functional Assessment of Cancer Therapy–Colorectal (FACT-C), [24]) or specific to the subgroups of these survivors, such as those with stoma (e.g., Modified City of Hope Quality of Life–Ostomy questionnaire (mCOH-QOL-O); [25]), and in their scope from the assessment of general QoL and its main domains (e.g., WHOQOL-BREF, [22]) to those assessing a specific aspect of QoL (e.g., QWLQ-CS, [23]). They also heavily differ in their development process, i.e., the theoretical underpinnings they are based on, and which kind of cancer survivor samples were included in the validation process, as well as in the information that is available regarding their psychometric properties [26]. Varied characteristics and therefore the usability of available questionnaires make the selection of the most appropriate tools quite complex, presenting another barrier to the integration of the assessment and its outputs meaningfully into practice by HCPs, on top of already existing barriers, such as their lack of time and knowledge [27]. Hence, the utilization of questionnaires designed to evaluate QoL domains corresponding to the primary concerns of HCPs in their clinical practice can facilitate the delivery of tailored interventions, enable effective monitoring of outcomes, and enhance the provision of personalized care, thereby optimizing their overall support for colorectal cancer survivors.

Therefore, the first aim of this study was to establish an expert consensus on the key domains of the QoL of colorectal cancer survivors that HCPs identify as important in clinical practice. The first research question (RQ1), i.e., What is the expert consensus regarding the important QoL domains of colorectal cancer survivors?, was addressed in a modified two-stage Delphi study in which HCPs evaluated already established domains and subdomains of QoL depicting cancer survivors’ perspectives. The second aim of the study was to review existing QoL questionnaires regarding their overlap between the domains assessed and the key domains identified in the Delphi study. The second research question (RQ2), i.e., What questionnaires exist for the assessment of QoL in colorectal cancer survivors and how do they correspond to the most important QoL domains by expert consensus?, was addressed in a scoping review study.

## 2. Study 1: Establishing Expert Consensus on Key Indicators of QoL in Colorectal Cancer Survivors

### 2.1. Materials and Methods

In Study 1, we followed a modified Delphi procedure which was previously described in detail in a study conducted on the QoL of breast cancer survivors [28]. The two-step procedure was adapted from related studies, such as Pietersma et al. [29] and Tung et al. [30]. In the first step, we identified a pool of domains and subdomains of QoL, and in the second, an interdisciplinary panel of experts participated in a two-round Delphi process, evaluating the importance of QoL (sub)domains in the care of colorectal cancer survivors.

#### 2.1.1. Identification of the Initial Pool of QoL Domains and Subdomains

The first phase of a Delphi methodology most often consists of a survey of expert opinions on the topic [31,32], or a survey is customized to better suit the research problem [33]. Following the latter and a similar approach used by Tung et al. [30], we chose a model of QoL in cancer survivors proposed by Ferrel and colleagues [34] and its variations [35,36,37] as the theoretical basis for the identification of the initial pool of QoL domains and subdomains. First, we mapped the subdomains of the models and identified overlapping ones and those specific to each model. Second, eight clinical experts and researchers of the research team of project [name of the project hidden for review] (Project Acronym; [38]) reviewed the list of extracted (sub)domains and added two subdomains often observed in clinical practice (in the physical domain, a subdomain of health distress, and in the psychological domain, loss of interest in usual activities). The final list consisted of four QoL domains (i.e., physical, psychological, social, and spiritual) with 35 subdomains, which represented the questionnaire items of the 1st round of the Delphi process.

#### 2.1.2. Evaluation of the Importance of (Sub)Domains

The second phase of Study 1 consisted of two rounds of evaluation of the importance of the identified (sub)domains and was conducted in spring and fall of 2020. Potential participants received an email invitation with an explanation of the study and a link to the online questionnaire. Informed consent was sought before participants filled out the questionnaire. Participants constructed their own unique IDs following the provided instructions, by which their responses were followed through both rounds. The study was performed in accordance with the Helsinki Declaration and its amendments, and ethical standards of the institutional and national research committees.

##### Participants

An adapted method of Borgiel and colleagues ([39], see also [30]) was applied for the recruitment of HCPs involved in the follow-up of colorectal cancer survivors, i.e., members of the research team of the project PERSIST, within which this study was conducted, recruited professional peers, i.e., contacts in their professional networks, to participate in the present study. In this phase, special attention was given to the recruitment of various HCP profiles (e.g., oncologists, physiotherapists, nurses, psychologists), from different European countries (i.e., Austria, Belgium, Latvia, Portugal, Slovenia, Spain, and Switzerland) due to the differences in healthcare systems and care paths for cancer survivors.

Delphi studies usually include the same experts in all survey rounds of the study, but this had to be modified in the present study, as many of the participants in the 1st round were not available to participate in the 2nd round due to the COVID-19 pandemic. Therefore, as only 16.67% of the participants of the 1st round participated in the 2nd round, we supplemented the sample of the 2nd round by inviting additional HCPs to participate following the same method as for the initial recruitment, with the aim of obtaining more representative results. Altogether, 70 HCPs participated in Study 1.

##### Questionnaire

The questionnaire for the 1st round consisted of 4 domains and 35 subdomains of QoL identified in the preparatory phase of this study. Participants rated the importance of the (sub)domains in the follow-up of the colorectal cancer survivors on a 7-point scale (1–not important; 7–very important). (Sub)domains that reached consensus in the 1st round were retained to be evaluated in the 2nd round, where the results of the 1st round in the form of the median answer and percentage of participants giving that answer were also presented. Again, participants rated the importance of the retained (sub)domains on a 7-point scale in the 2nd round.

##### Consensus Criterion and Analyses

The most common approach for establishing the criterion for consensus in Delphi studies is to specify a percentage level of agreement, which in existing studies varies from 51 to 100% [31,32]. Similar to Vanmeerbeek and colleagues [40] and Freitas and colleagues [41], we set the criterion at 75% in the present study. In the 1st round, consensus for each item was reached when at least 75% of participants agreed that the importance of the item merited one of the top three scores on the 7-point scale (i.e., they selected answers 5, 6, or 7), while in the 2nd round, consensus was reached when 75% of participants evaluated the item within the top two scores on the 7-point scale (i.e., they selected answers 6 or 7).

To assess the consistency of the participants’ ratings for each round, intraclass correlation coefficients (ICC) based on a mean-rating 2-way random effects model were calculated [42]. To assess the potential differences between the two groups of participants in the 2nd round (i.e., those who participated in the 1st round and new participants to the 2nd round), *t*-test results were calculated. Analyses were conducted in R version 4.1.1 ([43]; packages psych [44], and rstatix [45]).

### 2.2. Results

#### 2.2.1. Round 1

Fifty-four HCPs participated in the 1st round of the Delphi study (Table 1). Their inter-rater reliability was good [42] with ICC(2, 54) = 0.87, 95% CI [0.82, 0.90].

Consensus was reached for three out of four general domains (75%, i.e., the physical, psychological, and social domains; see Table 2), and for 31 out of 35 subdomains (88, 6%). Within the physical and psychological domains, all of the subdomains reached consensus (i.e., 10, 100.0% for both), while in the social domain, 8 out of 10 subdomains (80.0%) reached consensus. In the spiritual domain, none of the subdomains reached consensus in the 1st round.

#### 2.2.2. Round 2

In the 2nd round of the Delphi study, 25 HCPs participated (Table 1). Nine of them had participated in the 1st round (Group 1), while 16 participants were newly recruited to participate in the 2nd round only (Group 2). The reliability of all participants’ ratings was good [42] with ICC(2, 25) = 0.87, 95% CI [0.81, 0.92]. Independent sample *t*-tests (see Table 2) revealed no significant differences in the mean evaluations of the QoL (sub)domains between the two groups; therefore, we present the results on the reached consensus for all of the participants in the 2nd round together.

Consensus was reached for two out of three general domains (66.7%, i.e., the physical and psychological domains; see Table 2), and for 11 out of 31 subdomains (35.5%). Within the physical domain, 6 out of 10 subdomains reached consensus (60.0%); within the psychological domain, 3 out of 10 (30.0%); and within the social domain, none of the subdomains reached consensus in the 2nd round.

After two rounds of the Delphi process, the most important QoL (sub)domains for monitoring in the follow-up of colorectal cancer survivors by experts’ opinion were identified. On the level of the QoL domains, consensus was reached for the physical and psychological domains, and on the level of subdomains, the following were identified as most important: functional ability and mobility, activities of daily living, fatigue/vitality, pain and discomfort, physical symptoms, and physical health and comorbidities (all from the physical domain), and depression, psychological distress, and loss of interest in usual activities (from the psychological domain).

## 3. Study 2: Scoping Review of QoL Questionnaires for Colorectal Cancer Survivors

### 3.1. Materials and Methods

#### 3.1.1. Overview

Conducting this scoping review, the methodological framework by Arksey and O’Malley [47] and Levac and colleagues [48] was followed. It proposes six stages: (1) identifying the research question, (2) identifying the relevant studies, (3) study selection, (4) charting the data, (5) collating, summarizing, and reporting results, and (6) consultation exercises. Ensuring that the process was transparent, complete, and systematic, PRISMA-ScR (Preferred Reporting Items for Systematic Reviews and Meta-Analyses extension for Scoping Reviews [49]) was followed.

#### 3.1.2. Identifying the Research Questions

First, following the aim of the paper, we formulated specific research questions (RQs) to guide the scoping review:

- RQ2a: Which quality of life questionnaires for colorectal cancer survivors exist?
- RQ2b: What domains of quality of life do they assess?
- RQ2c: How do the identified domains of reviewed quality of life questionnaires overlap with the identified key indicators of quality of life by expert consensus?

#### 3.1.3. Identifying Relevant Studies

To identify relevant papers, three large and commonly used databases, i.e., SCOPUS, Web of Science, and PubMed, were used. We conducted a preliminary search in all three databases, helping us to refine the search strategy and ensuring that coverage of the topic was adequate. We performed the main search on 29 August 2022.

Our search strategy combined terms related to colorectal cancer (“colorectal cancer”, “colon cancer”), quality of life (“quality of life”, quality-of-life, well-being), questionnaires (questionnaire, scale, survey, instrument, “measurement tool”, “assessment tool”, assessment), psychometric validation (validation, psychometric), and survivorship (survivor*). We excluded all types of reviews (review, meta-analysis, “state of the art”, state-of-the-art) and limited our search to papers in English and published from the year 2000 forward to limit the results to more recent questionnaires. Listed groups of keywords were combined into a nested format using Boolean operators (AND, OR, NOT). Titles, abstracts, and keywords were searched. The final search string for SCOPUS was: “TITLE-ABS-KEY ((“colorectal cancer” OR “colon cancer”) AND (“quality of life” OR “quality-of-life” OR “well-being”) AND (questionnaire OR scale OR survey OR instrument OR “measurement tool” OR “assessment tool” OR “assessment”) AND (validation OR psychometric) AND (survivor *) AND NOT (review OR meta-analysis OR “state of the art” OR state-of-the-art)) AND LANGUAGE (english)) AND PUBYEAR > 1999”. We also performed an additional search in Google Scholar using different combinations of search terms, as this could lead to the identification of additional unique papers [50].

Inclusion and exclusion criteria were formulated on the basis of our research questions and were set a priori. These required studies to (1) have been published (i.e., excluding preprints and other unpublished papers), and (2) provide sufficient information regarding the questionnaire (at least dimensionality or subscales information), and required them not to (3) focus on any other constructs than quality of life (or health-related quality of life), (4) include questionnaires specific to other types of cancer (allowing for general cancer-related quality of life questionnaires), (5) be intended for use only with patients with cancer (rather than cancer survivors or both patients and survivors), and (6) focus on children or adolescents. Since the aim of this review was to review what QoL questionnaires for colorectal cancer survivors already exist and what they measure, we did not exclude primary studies based on their methodological quality [47].

#### 3.1.4. Identifying Relevant Studies

**Figure 1:**
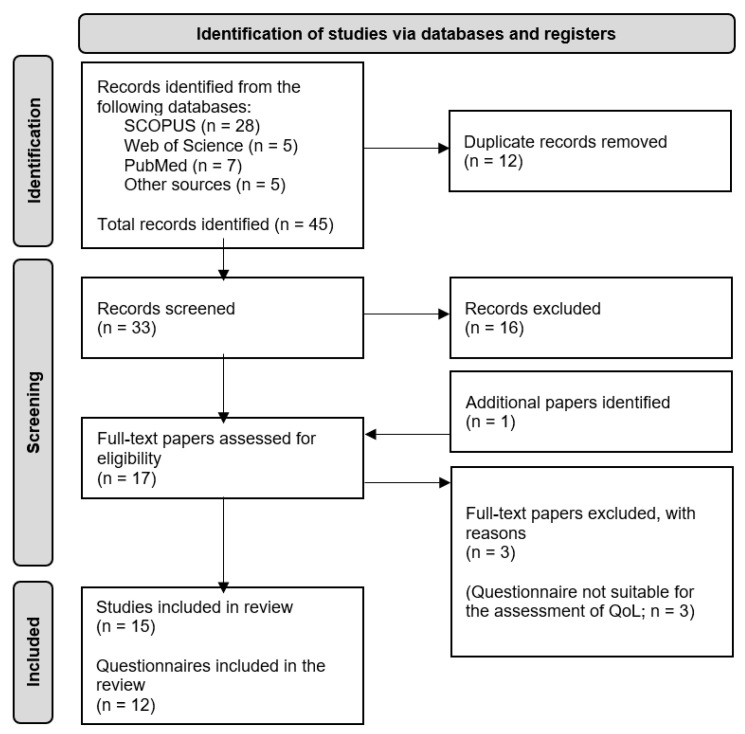
Search and study selection flowchart.

Citations of all the records identified (i.e., 45 papers, Figure 1) in the electronic databases were exported to Excel spreadsheets (Microsoft Inc). After duplicate records were removed, 33 titles and abstracts were screened independently by two authors (US and CL) to exclude irrelevant papers. After this step, two authors (US and CL) independently reviewed the full texts of the remaining papers and excluded papers not complying with the inclusion and exclusion criteria. Disagreements in each of the stages were settled through discussion. In the process of the full-text reviews, one additional eligible paper was identified. The process resulted in 15 papers containing information on 12 questionnaires that fulfilled the predetermined criteria and were included in the scoping review.

#### 3.1.5. Charting the Data

To chart the data, we formulated a spreadsheet based on the research questions, determining variables to be extracted from the reviewed papers. The following data were extracted from each paper: (1) authors, (2) year of publication, (3) type of paper (e.g., original questionnaire development/validation paper, questionnaire adaptation paper), (4) name of the questionnaire reported on in the paper, (5) what the questionnaire is intended to measure, (6) subscales/dimensions of the questionnaire and their descriptions, (7) number of items by subscale and in total, (8) response scale format, (9) focus population for the use of the questionnaire (e.g., cancer survivors in general, only colorectal cancer survivors), (10) languages of the questionnaire. The process of data extraction was completed by one author (SAC) and reviewed by another (US) in an iterative process during the review of the papers. The final chart was used for the analysis of the extracted information.

#### 3.1.6. Collating, Summarizing, and Reporting Results

After charting the data, information on questionnaires was summarized in order to address the first two research questions of this study, i.e., to identify what QoL questionnaires for colorectal cancer survivors exist and which domains of QoL they assess. Preparation of this part of the study was conducted by US.

#### 3.1.7. Consultation Exercises

The final step in the proposed methodological framework for scoping reviews [47,48] proposes (optional) consultation exercises with experts or stakeholders in the field. For the purpose of this study, the final step was adapted to address the third research question of this study, i.e., to explore how the identified domains in the reviewed QoL questionnaires for colorectal cancer survivors correspond to the key indicators of QoL identified in the Delphi study. This final step of the study was performed by US.

### 3.2. Results

#### 3.2.1. Characteristics of the Reviewed Questionnaires

The final selection of this review included 12 questionnaires that were identified in 15 studies (Table 3). In the following sections, we will be commenting only on questionnaires, not studies, with the exception of instances where studies provided differing information on the same questionnaire. The inclusion criteria for the papers and questionnaires to be included in the review stated that the questionnaires need to assess (health-related) quality of life. In the selection process, a slightly loose approach towards that criterion was taken, as there are several constructs that are heavily overlapping with the QoL construct. As such, the final selection consists of seven questionnaires assessing QoL [22,24,25,51,52,53,54,55,56,57,58,59,60,61], two assessing functional health and well-being [21,57,58], one well-being [24], one experienced burden and lifestyle parameters [62], and one quality of life in the work domain [23].

The target population of the questionnaires varies slightly among them. Most (i.e., 6 out of 12) of the questionnaires are intended to be used with adult patients with colorectal cancer (i.e., Assessment of Burden of Colorectal Cancer–tool (ABCRC), European Organisation for Research and Treatment of Cancer, Quality of Life Questionnaire for Colorectal Cancer-38 (EORTC QLQ-CR38), European Organisation for Research and Treatment of Cancer, Quality of Life Questionnaire for Colorectal Cancer-29 (EORTC QLQ-CR29), FACT-C, mCOH-QOL-O). Three of the questionnaires are intended to be used with adult patients and/or cancer survivors (i.e., European Organisation for Research and Treatment of Cancer, Quality of Life of Cancer Patients, version 3 (EORTC QLQ-C30), Quality of Life in Adult Cancer Survivors questionnaire (QLACS); QWLQ-CS), of which one (i.e., EORTC QlQ-C30) was also validated in the population of patients with colorectal cancer. Similarly, another questionnaire (i.e., European Organisation for Research and Treatment of Cancer, Quality of Life Questionnaire–Elderly Cancer Patients Module (EORTC QLQ-ELD14)) is intended to be used with ageing patients with cancer and has also been validated in a group of ageing patients with colorectal cancer. Among the reviewed questionnaires, there were three that were originally intended for assessing QoL in the general population and have also been validated in a population of patients with colorectal cancer (SF-36v2, SF-12v2 Health Survey (SF-12v2), WHOQOL-BREF). There were also two questionnaires, i.e., ABCRC and mCOH-QOL-O, suitable for patients with anastomosis and/or stoma, the first one for cancer patients, the latter for patients with or without cancer.

Other characteristics of the questionnaires, such as the number of items, the response scale, and the languages of the reviewed questionnaires, are provided in Table 3.

#### 3.2.2. Overview of the QoL Domains Assessed by the Reviewed Questionnaires

In this section, we provide an overview of the QoL domains addressed by the reviewed questionnaires and relate them to the key indicators of QoL that were identified in the Delphi study.

On the level of general domains (Table A1), six out of twelve questionnaires had specific subscales or items addressing this level of QoL. Three of them assess general QoL, seven the physical health and well-being domain, two psychological health and well-being, three social health and well-being, and two spiritual health and well-being. In the Delphi study on the level of general domains, physical and psychological health and well-being were identified as important indicators of QoL. The questionnaires that assess both domains are mCOH-QOL-O and WHOQOL-BREF. Additionally, several questionnaires assess only physical health and well-being among the identified key indicators, i.e., EORTC QLQ-C30, FACT-C, mCOH-QOL-O, SF-36v2, SF-12v2 and WHOQOL-BREF.

In the following text, we focus on the overview of subdomains of QoL. Within the physical domain (Table A2), nine questionnaires assess at least one of the subdomains (or a part of a subdomain) included in Study 1, while eight questionnaires also assess other subdomains of physical health and well-being outside those of Study 1. Focusing first on the subdomains identified in the Delphi study as key indicators, two questionnaires assess functional ability and mobility (i.e., ABCRC and EORTC QLQ-ELD14), one activities of daily living (i.e., ABCRC), five fatigue/vitality (i.e., ABCRC, EORTC QLQ-C30, QLACS, SF-36v2, SF-12v2), five pain and discomfort (i.e., EORTC QLQ-C30, EORTC QLQ-CR29, QLACS, SF-36v2, SF-12v2), four physical symptoms (i.e., ABCRC, EORTC QLQ-C30, EORTC QLQ-CR38, EORTC QLQ-CR29), and none of them assess physical health and comorbidities. While none of the questionnaires assess all six subdomains identified as key indicators in the Delphi study, most of them, i.e., four, are assessed by the ABCRC questionnaire. Other questionnaires assess one or two of the key subdomains. Additionally, some questionnaires also assess other physical health and well-being subdomains that were not identified as key indicators. These are sleep and rest, assessed by one questionnaire; health perceptions, assessed by two questionnaires; and weight loss/gain, assessed by one questionnaire.

Within the psychological domain (Table A3), three subdomains were identified as key indicators in the Delphi 1 study, i.e., depression, psychological distress, and loss of interest in usual activities. Among the reviewed questionnaires, only one assesses at least one of these subdomains, i.e., the EORTC QLQ-ELD14 questionnaire that assesses psychological distress. Other questionnaires assess subdomains that were not identified as key indicators: anxiety is assessed by one questionnaire, cognitive functioning and concentration by three, and other subdomains considered by only one of the questionnaires include uncertainty, fear of recurrence, isolation/abandonment and feelings of belonging, positive feelings and affect, and negative feelings and affect. Six questionnaires also assessed other subdomains that were not included in Study 1.

Within the social domain (Table A4), none of the subdomains were identified as key indicators in the Delphi study. Among other subdomains, family functioning is assessed by two questionnaires, marital functioning by one, affection/sexuality by none, self-conception/appearance by four, enjoyment/leisure (participation and opportunities) by none, social activity and limitations by one, financial concerts by three, social support by none, employment by two, and role limitations due to health or physical problems by three questionnaires. Five questionnaires also assess other subdomains that were not included in Study 1.

Within the spiritual domain (Table A5), none of the subdomains were identified as key indicators in the Delphi study. Also, none of the reviewed questionnaires assess any of the subdomains.

The reviewed questionnaires also assess other (sub)domains of QoL that we were not able to classify under any of general domains, as presented in Table A6.

## 4. Discussion

With the growing population of colorectal cancer survivors [8], the focus on their clinical care and support is shifting towards the recognition of the importance of their QoL [16,17], highlighting a need for regular assessments and monitoring of QoL that are necessary to detect areas that might need to be addressed for each individual patient. Even though several barriers to the implementation of such assessments in the clinical routine persist [27], identifying the QoL domains that are of the utmost importance not only to the survivors but also to their HCPs, along with providing appropriate questionnaires that address those domains, is critical to assist this process. Therefore, in the present study, we established an expert consensus on the key domains of the QoL of colorectal cancer survivors in a modified Delphi study and reviewed existing QoL questionnaires for this population regarding their correspondence to the identified key QoL domains.

In the Delphi study, expert consensus regarding the QoL general domains was reached for the physical and psychological domains. HCPs also evaluated QoL subdomains where consensus was reached for the following subdomains of the physical domain: functional ability and mobility, activities of daily living, fatigue/vitality, pain and discomfort, physical symptoms, and physical health and comorbidities. Consensus was also reached for the following subdomains of the psychological domain: depression, psychological distress, and loss of interest in usual activities. In the scoping review of existing QoL questionnaires, 12 of them were identified, and the domains that they assessed were mapped onto the list of (sub)domains that were evaluated by HCPs in the Delphi study. On the level of general domains, only two questionnaires assess both domains that were identified as important by HCPs, i.e., mCOH-QOL-O [25] and WHOQOL-BREF [22]. On the level of the physical QoL subdomains, none of the questionnaires address all the key subdomains, but most of them, i.e., four out of six, are covered by the ABCRC questionnaire [62], while some other questionnaires assess one or two key subdomains (see Table A1). On the level of the psychological QoL subdomains, no questionnaire addressed all three key subdomains and only one, i.e., EORTC QLQ-ELD14 [56], addressed one of these three subdomains. On the level of the social and spiritual QoL subdomains, consensus was reached for none of them in the Delphi study; however, several of the questionnaires address the social subdomains, but none address the spiritual. It is also important to note that most of the reviewed questionnaires also assess several other (sub)domains that were not identified as important in the clinical routines of the HCPs in our study, which does not mean that they are not important for the survivors or in some other contexts.

Following the results of this study, we can conclude that two questionnaires, mCOH-QOL-O [25], and WHOQOL-BREF [22], address both key general domains, and on the level of subdomains, ABCRC [62] and EORTC QLQ-ELD14 [56] assess the most of those identified as key by HCPs, while none of them address them all. Even though there is a lot of room left for improvement in the assessment of cancer survivors’ QoL [12], the reason for the relatively low correspondence between HCPs’ identified key QoL domains and domains addressed by the reviewed questionnaires could lie in the fact that in the Delphi study involved various profiles of HCPs. As these results represent a wide and general consensus among several professional profiles that are in contact with colorectal cancer survivors in clinical routine, it would be worthwhile to explore whether the key domains identified differ among these profiles, which may reflect specific focuses regarding survivors’ health aspects.

Nevertheless, questionnaires that provide the information most needed by HCPs regarding survivors’ QoL are important. Beyond the QoL domains that they provide information on, they of course need to be psychometrically validated and standardized, which was not the focus of the present study. However, metric characteristics should be evaluated before any of these questionnaires is implemented [12]. For instance, of the two questionnaires that address both key general domains, WHOQOL-BREF is provided with a plethora of studies and evidence in support of its psychometric quality in several languages and populations [22,63,64,65], while mCOH-QOL-O is much less supported in this regard. In both cases, however, additional studies on the population of colorectal cancer survivors would substantially benefit their justified use in this population.

High-quality questionnaires that address the domains that are of the highest importance for both survivors and HCPs can aid in providing appropriate care and support in the areas needed, especially if they can be integrated in the clinical routine. However, the appropriateness of most of the reviewed questionnaires for frequent assessments is questionable, i.e., the questionnaires contain from 27 up to 107 items (except for EORTC QLQ-ELD14 and SD-12v2 which contain 14 and 12 items, respectively), and as such present an additional burden for the survivors if requested to be filled in regularly. Therefore, before integration into regular assessment, developing shorter but still telling versions of appropriate questionnaires would be beneficial as they would have a strong potential to be employed in evaluating the outcomes of colorectal cancer survivors [66]. Such efforts might be additionally supported by other advances in the field, e.g., prediction models of individuals with heightened risk of lowered QoL levels [8], adaptive approaches to assessing QoL dimensions [67], and even research in quality measurements in healthcare [68].

Such advancements, combined with the current research including the present study, hold several implications for the research and practice of QoL assessment in colorectal cancer survivors. Our study underscores the disparity between a framework of crucial QoL domains identified by survivors [34,35,36,37] and those identified by HCPs, and the availability of tools with sufficient empirical evidence supporting their validity. Therefore, on top of using appropriate instruments, we recommend long-term assessment of QoL due to the frequent experience of symptoms that affect QoL well into the survivorship period [69], perhaps with special attention paid to the most important QoL (sub)domains identified in this study. However, QoL needs to be considered in a way that accounts for demographic and clinical factors, such as age, diagnosis specifics, potential comorbidities, and the individual needs of survivors, specifically in clinical practice, as these can significantly impact the levels of QoL colorectal cancer survivors experience and report [69].

### Limitations of the Study

While the current paper offers a valuable contribution to the area of assessment of the QoL of colorectal cancer survivors, its findings are limited for the following reasons. The Delphi procedure employed in the first study deviated from the standard Delphi methodology [31,32] in the sample of participants involved, as there was relatively low overlap in the samples of both rounds. However, since the outcomes of Delphi studies do not necessarily represent the opinions of all experts due to relatively small sample sizes [30], the inclusion of additional experts in the second round could aid in achieving a higher-level of generalizability. This is additionally supported by the inclusion of international and multidisciplinary samples. Therefore, our results represent a consensus on key QoL domains in the clinical routines of various HCP profiles. To establish a consensus specific to each HCP profile involved in the clinical care of colorectal cancer survivors, further studies are needed. Similarly, as age and length of survivorship are important variables in the QoL of cancer survivors [70], the specifics in the most important QoL domains to be monitored may also vary depending on these factors, so further studies are needed. In the scoping review of current QoL questionnaires for colorectal cancer survivors, we did not assess the psychometric quality of these questionnaires [12]. Therefore, for each of the questionnaires discussed in this paper, researchers/HCPs need to explore their characteristics before employing them in their work with colorectal cancer survivors.

## 5. Conclusions

The first part of the current study established an expert consensus on the most important QoL domains of colorectal cancer survivors in clinical practice from the HCPs’ perspective via a two-round process of a Delphi study. The results show that consensus was reached on the physical and psychological domains, and on 17 of 30 subdomains, all within the physical and psychological domains. In the second part, i.e., the scoping review, 12 QoL questionnaires were identified and reviewed regarding their correspondence to the domains identified in the Delphi study. On the level of general domains, there are two questionnaires that assess the physical and psychological domains. On the level of subdomains, none of the questionnaires assess all the key domains established in the Delphi study, but several of them are appropriate for assessing a few of these subdomains. The findings of this study clearly point to the relative mismatch between currently available assessment tools and the areas HCPs are most interested in during their clinical work with colorectal cancer survivors. Therefore, this study may serve as an indicator of what kinds of questionnaires would serve HCPs better, which could also aid in the process of integration of routine QoL assessments in monitoring the colorectal cancer survivors, to enhance the efforts of healthcare to aid and support survivors’ QoL.

## Figures and Tables

**Table 1:** Participants’ characteristics.

		Round 1		Round 2					
				All Participants		Group 1		Group 2	
		f	%	f	%	f	%	f	%
N		54		25		9		16	
Gender	Female	33	61.1	16	64.0	4	44.4	12	75.0
	Male	21	38.9	9	36.0	5	55.6	4	25.0
Speciality *	Gastroenterology	4	7.4	1	4.0	-	-	1	6.3
	Medical Oncology	16	29.6	9	36.0	4	44.4	5	31.3
	Nutrition	2	3.7	2	8.0	1	11.1	1	6.3
	Oncology Nursing	5	9.3	-	-	-	-	-	-
	Physiotherapy	1	1.9	4	16.0	-	-	4	25.0
	Psychology	5	9.3	-	-	-	-	-	-
	Psychotherapy	-	-	-	-	-	-	-	-
	Radiology & Radiotherapy Oncology	3	5.6	2	8.0	2	22.2	-	-
	Surgery	8	14.8	5	20.0	2	22.2	3	18.8
	Other	11	20.4	3	12.0	1	11.1	2	12.5
Country	Austria	-	-	3	12.0	-	-	3	18.8
	Belgium	10	18.5	10	40.0	3	33.3	7	43.8
	Latvia	6	11.1	5	20.0	3	33.3	2	12.5
	Portugal	22	40.7	2	8.0	-	-	2	12.5
	Slovenia	2	3.7	3	12.0	2	22.2	1	6.3
	Spain	13	24.1	1	4.0	1	11.1	-	-
	Switzerland	1	1.9	1	4.0	-	-	1	6.3
Years in practice	M	15.7		10.2		12.2		9.1	
	SD	10.3		8.3		9.7		7.6	

Notes. Group 1: participants included in both rounds; Group 2: participants included only in the 2nd round. * In Speciality for Round 2, the frequencies do not add up to exact N of Group 1 and consequently to All participants of Round 2, as one participant indicated two specialities, i.e., Medical oncology and Surgery.

**Table 2:** Delphi Rounds 1 and 2–QoL (sub)domains, mean ratings with standard deviations and consensus.

	Round 1				Round 2										
					All Participants		Group 1 ^c^		Group 2 ^d^		*t*-Test ^e^				
	M	SD	% Agreement ^a^	CR ^b^	M	SD	M	SD	M	SD	df	*t*	Adj. *p* ^f^	% Agreement ^g^	CR ^h^
**General domains**															
Physical Health and Well-being	6.3	1.0	94.4	*	6.3	0.5	6.1	0.3	6.4	0.5	23	−1.41	0.87	100.0	*
Psychological Health and Well-being	6.0	1.0	94.4	*	6.3	0.8	6.1	0.8	6.4	0.8	23	−0.79	0.87	80.0	*
Social Health and Well-being	5.6	1.0	83.3	*	5.8	0.9	5.7	0.5	5.9	1.0	23	−0.57	0.88	68.0	-
Spiritual Health and Well-being	4.9	1.6	61.1	-	-	-	-	-	-	-	-	-	-	-	-
**Physical QoL**															
Functional ability and Mobility	6.2	0.9	96.0	*	6.5	0.7	6.7	0.5	6.5	0.8	20	0.70	0.87	90.9	*
Activities of daily living	6.1	0.9	94.0	*	6.6	0.6	6.7	0.5	6.6	0.7	20	0.20	0.94	95.5	*
Fatigue/Vitality	5.9	0.8	96.0	*	6.0	0.6	6.2	0.4	5.9	0.6	20	1.21	0.87	95.5	*
Sleep and rest	5.7	1.1	90.0	*	5.7	0.8	5.4	0.7	5.9	0.9	20	−1.36	0.87	68.2	-
Pain and discomfort	6.4	0.9	94.0	*	7.0	0.2	7.0	0.0	6.9	0.3	20	0.83	0.87	100.0	*
Health perceptions	5.6	0.9	90.0	*	5.3	0.8	5.2	0.4	5.4	1.0	20	−0.47	0.91	31.8	-
Physical symptoms	6.2	0.8	98.0	*	6.4	0.5	6.4	0.5	6.3	0.5	20	0.63	0.87	100.0	*
Health distress	5.7	1.0	94.0	*	5.3	0.8	5.0	0.7	5.5	0.9	20	−1.31	0.87	31.8	-
Weight loss/gain	6.0	0.9	92.0	*	5.8	1.0	6.3	0.7	5.5	1.0	20	2.30	0.50	68.2	-
Physical Health and comorbidities	6.0	0.9	94.0	*	5.9	0.8	5.8	0.8	5.9	0.9	20	−0.39	0.92	77.3	*
**Psychological QoL**															
Anxiety	5.8	1.1	88.0	*	5.7	1.0	5.7	1.1	5.8	0.9	19	−0.19	0.94	66.7	-
Depression	5.9	1.0	90.0	*	6.0	0.9	6.1	1.1	5.8	0.8	19	0.67	0.87	76.2	*
Psychological distress	5.8	1.1	84.0	*	6.0	0.7	6.1	0.8	6.0	0.7	19	0.33	0.92	76.2	*
Cognitive functioning, concentration and attention	5.5	1.3	82.0	*	5.5	1.0	5.3	1.2	5.6	0.9	19	−0.54	0.88	52.4	-
Uncertainty	5.2	1.1	76.0	*	5.0	0.9	5.0	0.7	5.1	1.0	19	−0.21	0.94	28.6	-
Fear of Recurrence	5.6	1.0	86.0	*	5.8	0.8	5.8	1.0	5.8	0.7	19	−0.15	0.94	66.7	-
Isolation/Abandonment and feelings of belonging	5.5	1.2	82.0	*	5.6	0.9	5.0	0.9	6.0	0.6	19	−3.13	0.17	61.9	-
Positive feelings and affect	5.6	1.0	88.0	*	5.8	0.8	5.4	0.7	6.0	0.7	19	−1.72	0.87	66.7	-
Negative feelings and affect	5.5	1.1	86.0	*	5.8	0.5	5.7	0.5	5.8	0.6	19	−0.69	0.87	71.4	-
Loss of interest in usual activities	5.5	1.3	80.0	*	6.0	0.8	5.8	1.2	6.2	0.4	19	−1.06	0.87	90.5	*
**Social QoL**															
Family functioning	5.7	1.2	78.0	*	5.9	1.1	6.1	1.1	5.7	1.3	17	0.77	0.87	68.4	-
Marital functioning	5.2	1.4	72.0	-	-	-	-	-	-	-	-	-	-	-	-
Affection/Sexuality	5.2	1.3	68.0	-	-	-	-	-	-	-	-	-	-	-	-
Self-Conception/Appearance	5.4	1.2	78.0	*	5.3	0.7	5.3	0.5	5.3	0.9	17	0.09	0.94	36.8	-
Enjoyment/Leisure (participation and opportunities)	5.4	1.3	78.0	*	5.7	0.9	5.7	0.9	5.7	1.1	17	−0.07	0.94	57.9	-
Social activity and limitations	5.6	1.2	80.0	*	5.8	0.8	5.8	0.4	5.9	1.0	17	−0.34	0.92	73.7	-
Financial Concerns	5.2	1.3	76.0	*	4.8	1.1	4.7	0.7	5.0	1.3	17	−0.67	0.87	21.1	-
Social Support	5.5	1.1	84.0	*	5.5	0.7	5.3	0.7	5.7	0.7	17	−1.16	0.87	63.2	-
Employment	5.5	1.2	82.0	*	5.7	0.7	5.6	0.5	5.8	0.8	17	−0.78	0.87	57.9	-
Role limitations due to health or psychical problems	5.4	1.2	78.0	*	5.5	0.6	5.3	0.5	5.7	0.7	17	−1.33	0.87	47.4	-
**Spiritual QoL**															
Meaning of Illness	5.2	1.5	72.0	-	-	-	-	-	-	-	-	-	-	-	-
Religiosity	4.1	1.9	48.0	-	-	-	-	-	-	-	-	-	-	-	-
Hope	5.2	1.4	68.0	-	-	-	-	-	-	-	-	-	-	-	-
Transcendence	4.4	1.6	50.0	-	-	-	-	-	-	-	-	-	-	-	-
Inner Strength	5.0	1.5	64.0	-	-	-	-	-	-	-	-	-	-	-	-

Notes. ^a^ % of all participants evaluating the item with the top three measures on a 7-point scale. ^b^ * = consensus reached (criterion: % Agreement for round 1 < 75%). ^c^ Group 1: Participants included in both Delphi rounds. ^d^ Group 2: Participants included only in the 2nd Delphi round. ^e^ Independent samples *t*-test, 2-tailed, equal variances assumed. ^f^ p adjustment method = BH [46]. ^g^ % of all participants evaluating the item with top two measures on a 7-point scale. ^h^ * = consensus reached (criterion. % Agreement for round 2 < 75%), - = consensus not reached.

**Table 3:** An overview of the characteristics of the papers and studies identified.

Questionnaire	Reference	Type of Paper	Construct Assessed	Target Population	Number of Items	Response Scale	Language of the Questionnaire
ABCRC (Assessment of Burden of Colorectal Cancer-tool)	Boome et al., 2022 [62]	Development, content validation	Experienced burden of colorectal cancer and lifestyle parameters	Adult patients with colon and rectal cancer, patients with anastomosis, and patients with stoma	27 (version for patients with stoma), 28 (version for colon cancer), 29 (version for rectal cancer)	3- and 4-point; one open question	Dutch
EORTC QLQ-C30 (European Organisation for Research and Treatment of Cancer, Quality of Life of Cancer Patients, version 3)	Calderon et al., 2022 [51]	Validation	Quality of life	Adult patients with cancer (general)	30	4-point Likert type and 7-point	Spanish
	El Alami et al., 2021 [52]	Validation (Moroccan Arabic Version)		Adult patients with cancer (general); in this study colorectal cancer patients			Moroccan Arabic
EORTC QLQ-CR38 (European Organisation for Research and Treatment of Cancer, Quality of Life Questionnaire for Colorectal Cancer-38) ^1^	Rotonda et al., 2008 [24]	Validation (French version)	Quality of life	Adult patients with colorectal cancer	38	4-point Likert type and 7-point	French
EORTC QLQ-CR29 (European Organisation for Research and Treatment of Cancer, Quality of Life Questionnaire for Colorectal Cancer-29) ^1,2^	Gujral et al., 2007 [53]	Content validation study (adaptation of the EORTC QLQ-CR38)	Quality of life	Adult patients with colorectal cancer	29	[no information available in the paper]	English
	Al-Shandudi et al., 2022 [54]	Empirical study					Arabic
	Whistance et al., 2009 [55]	Clinical and psychometric validation				4-point Likert type and 7-point	English, French, Taiwanese, Italian, German, Spanish
EORTC QLQ-ELD14 (European Organisation for Research and Treatment of Cancer, Quality of Life Questionnaire-Elderly Cancer Patients Module)	Lorca et al., 2021 [56]	Validation for ageing cancer survivors	Quality of life	Ageing patients (>60 years) with cancer (in this study colorectal cancer survivors)	14	7-point Likert scale and 4-point scale	Spanish version
FACT-C (Functional Assessment of Cancer Therapy–Colorectal)	Rotonda et al., 2008 [24]	Validation	Well-being	Adult patients with colorectal cancer	34	5-point scale	French
mCOH-QOL-O (Modified City of Hope Quality of Life-Ostomy questionnaire)	Grant et al., 2004 [25]	Validation	Quality of life	Ostomy patients (cancer and non-cancer)	41	11-point scale	English
	Wendel et al., 2014 [57]			Rectal cancer survivors	43		
	Mohler et al., 2008 ^3^ [58]	Empirical paper		Colorectal cancer (CRC) survivors with and withoutostomies	34		English
QLACS (Quality of Life in Adult Cancer Survivors questionnaire)	Avis et al., 2005 [59]	Questionnaire development	Quality of life	Long-term cancer survivors (> or equal to 5 years post-diagnosis)	47	7-point scale	English
	Ashley et al., 2014 [60]	Psychometric validation		Short-term cancer survivors			
	Escobar et al., 2015 [61]	Cross-cultural adaptation, reliability andvalidity of the Spanish version					Spanish
QWLQ-CS (Quality of Working Life Questionnaire for Cancer Survivors)	De Jong et al., 2016 ^4^ [23]	Item-development study	Quality of life in the work domain	Cancer survivors	104	4-point scale	English
SF-36v2 (SF-36v2 Health Survey)	Ware & Sherbourne, 1992 [21]	Item selection study	Functional health and well-being	General population	36	several types of response scales differing by items	English
	Mohler et al., 2008 [58]	Empirical study					
SF-12v2 (SF-12v2 Health Survey)	Wendel et al., 2014 [57]	Validation paper–for use with long-term rectal cancer survivors	Functional health and well-being	[no information]	12	[no information]	English
WHOQOL-BREF (World Health Organization Quality-of-Life Scale)	Lin et al., 2019 [22]	Psychometric validation of Taiwan version	Quality of life	General population (sample of this study: cancer survivors, also colorectal)	28 (original 26 items + 2 added in this study)	5-point scale	Taiwanese

Notes. ^1^ To be used with the core questionnaire EORTC QlQ-C30. ^2^ Additionally included paper, since Al-Shandudi et al. [54] referred to it regarding the questionnaire. ^3^ A modified version of the questionnaire (omitted ostomy-specific items) was used with control subjects in the study. ^4^ Not a validation paper, but only an item-generation study paper, resulting in a preliminary questionnaire without tested psychometric properties.

## Data Availability

The dataset used and analyzed during the current study is available from the corresponding author upon reasonable request.

## Appendix A

**Table A1:** Questionnaires’ QoL areas of assessment and key QoL indicators–general QoL domains.

QoL Domain	Consensus Reached in Study 1	ABCRC	EORTC QLQ-C30	EORTC QLQ-CR38	EORTC QLQ-CR29		EORTC QLQ-ELD14	FACT-C	mCOH-QOL-O		QLACS	QWLQ-CS	SF-36v2	SF-12v2	WHOQOL-BREF
					A	B			A	B					
Physical Health and Well-being	*		X					X	X	X			X	X	X
Psychological Health and Well-being	*								X	X					X
Social Health and Well-being								X		X					X
Spiritual Health and Well-being									X	X					
*General QoL*			X	X						X					
**QoL areas assessed**															
… of all domain areas of Study 1 (4 + 1 general QoL))			2	1				2	3	5			1	1	3
… of areas of consensus reached in Study 1 [2]			1					1	2	2			1	1	2

Notes. Questionnaires’ references: ABCRC [62]; EORTC QLQ-C30 [51,52] M; EORTC QLQ-CR38 [24]; EORTC QLQ-CR29 ver. A [53,54]; EORTC QLQ-CR29 ver. B [55]; EORTC QLQ-ELD14 [56]; FACT-C [24]; mCOH-QOL-O ver. A [25]; mCOH-QOL-O ver. B [57,58]; QLACS [59,60,61]; QWLQ-CS [23]; SF-36v2 [21,58]; SF-12v2 [57]; WHOQOL-BREF [22]. X marks the questionnaire addresses the (sub)domain. * marks that consensus had been reached among experts.

**Table A2:** Questionnaires’ QoL areas of assessment and key QoL indicators–physical QoL domain.

QoL Domain	Consensus Reached in Study 1	ABCRC	EORTC QLQ-C30	EORTC QLQ-CR38	EORTC QLQ-CR29		EORTC QLQ-ELD14	FACT-C	mCOH-QOL-O		QLACS	QWLQ-CS	SF-36v2	SF-12v2	WHOQOL-BREF
					A	B			A	B					
Functional ability and Mobility	*	X					X								
Activities of daily living	*	X													
Fatigue/Vitality	*	X	X								X		X	X	
Sleep and rest			X												
Pain and discomfort	*		X		X						X		X	X	
*Abdominal pain*						X									
*Buttock pain*						X									
Health perceptions													X	X	
Physical symptoms	*	X													
*General gastrointestinal*		X		X											
*Hair loss*					X	X									
*Dry mouth*					X	X									
*Dyspnea*			X												
*Taste problems*					X	X									
*Nausea, vomiting*			X												
*Skin problems*					X	X									
*Urinary problems*					X										
*Urinary frequency*						X									
*Urinary incontinence*						X									
*Painful urination*						X									
*Stool frequency*						X									
*Defecation problems*				X	X										
*Stool irregularities*						X									
*Constipation*			X												
*Diarrhea*			X												
*Faecal incontinence*					X	X									
*Intoxications*		X													
*Bloated abdomen*					X	X									
*Flatulence*						X									
Health distress															
Weight loss/gain				X		X									
Physical Health and comorbidities	*														
Other in physical domain															
*Appetite, nutrition*		X	X												
*Sexual enjoyment*				X											
*Sexual functioning*				X											
*Sexual problems*											X				
*Sexual dysfunction, impotence*				X	X	X									
*Sexual interest*					X	X									
*Dysapreunia*					X	X									
*Disease-specific effects on physical well-being*									X						
*Change in health*													X		
**QoL areas assessed**															
… of all domain areas of Study 1 [10]		4	3	1	2	2	1				2		3	3	
… of areas of consensus reached in Study 1 [6]		4	2		1	1	1				2		2	2	
… of ‘Other’ domain areas [9]		1	1	3	3	3			1		1		1		

Notes. Italic QoL areas are the ones additionally identified in the questionnaires of Study 2 but not present in Study 1 QoL area pool. Questionnaires’ references: ABCRC [62]; EORTC QLQ-C30 [51,52] M; EORTC QLQ-CR38 [24]; EORTC QLQ-CR29 ver. A [53,54]; EORTC QLQ-CR29 ver. B [55]; EORTC QLQ-ELD14 [56]; FACT-C [24]; mCOH-QOL-O ver. A [25]; mCOH-QOL-O ver. B [57,58]; QLACS [59,60,61]; QWLQ-CS [23]; SF-36v2 [21,58]; SF-12v2 [57]; WHOQOL-BREF [22]. X marks the questionnaire addresses the (sub)domain. * marks that consensus had been reached among experts.

**Table A3:** Questionnaires’ QoL areas of assessment and key QoL indicators–psychological QoL domain.

QoL Domain	Consensus Reached in Study 1	ABCRC	EORTC QLQ-C30	EORTC QLQ-CR38	EORTC QLQ-CR29		EORTC QLQ-ELD14	FACT-C	mCOH-QOL-O		QLACS	QWLQ-CS	SF-36v2	SF-12v2	WHOQOL-BREF
					A	B			A	B					
Anxiety					X	X									
Depression	*														
Psychological distress	*						X								
Cognitive functioning, concentration and attention		X	X								X				
Uncertainty				X											
Fear of Recurrence											X				
Isolation/Abandonment and feelings of belonging															
Positive feelings and affect											X				
Negative feelings and affect											X				
Loss of interest in usual activities	*														
Other in psychological domain															
General mental health													X	X	
Emotional functioning and well-being		X	X					X							
Disease-specific effects on psychological well-being									X						
QoL areas assessed															
… of all domain areas of Study 1 [10]		1	1	1	1	1	1				4				
… of areas of consensus reached in Study 1 [3]							1								
… of ‘Other’ domain areas [3]		1	1					1	1				1	1	

Notes. Italic QoL areas are the ones additionally identified in the questionnaires of Study 2 but not present in Study 1 QoL area pool. Questionnaires’ references: ABCRC [62]; EORTC QLQ-C30 [51,52] M; EORTC QLQ-CR38 [24]; EORTC QLQ-CR29 ver. A [53,54]; EORTC QLQ-CR29 ver. B [55]; EORTC QLQ-ELD14 [56]; FACT-C [24]; mCOH-QOL-O ver. A [25]; mCOH-QOL-O ver. B [57,58]; QLACS [59,60,61]; QWLQ-CS [23]; SF-36v2 [21,58]; SF-12v2 [57]; WHOQOL-BREF [22]. X marks the questionnaire addresses the (sub)domain. * marks that consensus had been reached among experts.

**Table A4:** Questionnaires’ QoL areas of assessment and key QoL indicators–social QoL domain.

QoL Domain	Consensus Reached in Study 1	ABCRC	EORTC QLQ-C30	EORTC QLQ-CR38	EORTC QLQ-CR29		EORTC QLQ-ELD14	FACT-C	mCOH-QOL-O		QLACS	QWLQ-CS	SF-36v2	SF-12v2	WHOQOL-BREF
					A	B			A	B					
Family functioning		X									X				
Martial functioning		X													
Affection/Sexuality															
Self-Conception/Appearance				X	X	X					X				
Enjoyment/Leisure (participation and opportunities)															
Social activity and limitations											X				
Financial Concerns		X	X								X				
Social Support															
Employment		X										X			
*… general feelings about working life*												X			
*… job characteristics*												X			
*… social structure and culture*												X			
*… contact with supervisor*												X			
*… contact with other actors at work*												X			
*… organisational characteristics*												X			
*… work perceptions*												X			
*… effects of the disease and treatment (on work)*												X			
Role limitations due to health or psychical problems			X												
*… due to physical problems*													X	X	
*… due to emotional problems*													X	X	
*Other in social domain*															
*Social adjustment to ostomy*									X						
*Social functioning*			X										X	X	
QoL areas assessed															
… of domain areas of Study 1 [10]		4	2	1	1	1					4	1	1	1	
… of ‘Other’ domain areas [2]			1						1			8	1	1	

Notes. Italic QoL areas are the ones additionally identified in the questionnaires of Study 2 but present in Study 1 QoL area pool. Questionnaires’ references: ABCRC [62]; EORTC QLQ-C30 [51,52] M; EORTC QLQ-CR38 [24]; EORTC QLQ-CR29 ver. A [53,54]; EORTC QLQ-CR29 ver. B [55]; EORTC QLQ-ELD14 [56]; FACT-C [24]; mCOH-QOL-O ver. A [25]; mCOH-QOL-O ver. B [57,58]; QLACS [59,60,61]; QWLQ-CS [23]; SF-36v2 [21,58]; SF-12v2 [57]; WHOQOL-BREF [22]. X marks the questionnaire addresses the (sub)domain.

**Table A5:** Questionnaires’ QoL areas of assessment and key QoL indicators–spiritual QoL domain.

QoL Domain	Consensus Reached in Study 1	ABCRC	EORTC QLQ-C30	EORTC QLQ-CR38	EORTC QLQ-CR29		EORTC QLQ-ELD14	FACT-C	mCOH-QOL-O		QLACS	QWLQ-CS	SF-36v2	SF-12v2	WHOQOL-BREF
					A	B			A	B					
SPIRITUAL QOL															
Meaning of Illness															
Religiosity															
Hope															
Transcendence															
Inner Strength															
QoL areas assessed															
… of domain areas of Study 1 [5]															

Notes. Italic QoL areas are the ones additionally identified in the questionnaires of Study 2 but not present in Study 1 QoL area pool. Questionnaires’ references: ABCRC [62]; EORTC QLQ-C30 [51,52] M; EORTC QLQ-CR38 [24]; EORTC QLQ-CR29 ver. A [53,54]; EORTC QLQ-CR29 ver. B [55]; EORTC QLQ-ELD14 [56]; FACT-C (24); mCOH-QOL-O ver. A (25); mCOH-QOL-O ver. B [57,58]; QLACS [59,60,61]; QWLQ-CS [23]; SF-36v2 [21,58]; SF-12v2 [57]; WHOQOL-BREF [22].

**Table A6:** Questionnaires’ QoL areas of assessment and key QoL indicators–other QoL areas.

QoL Domain	Consensus Reached in Study 1	ABCRC	EORTC QLQ-C30	EORTC QLQ-CR38	EORTC QLQ-CR29		EORTC QLQ-ELD14	FACT-C	mCOH-QOL-O		QLACS	QWLQ-CS	SF-36v2	SF-12v2	WHOQOL-BREF
					A	B			A	B					
Cancer-specific scale								X							
Treatment-related symptoms				X											
Stoma-related symptoms etc.		X		X	X	X									
Embarrassment (by stoma, bowel movement, …)					X	X									
Environment domain															X
Disease burden							X								
Benefits of cancer											X				
Functional well-being								X							
Maintenance purposes							X								
Private life												X			
Other QoL areas assessed		1		2	2	2	2	2			1	1			1

Notes. Italic QoL areas are the ones additionally identified in the questionnaires of Study 2 but not present in Study 1 QoL area pool. Questionnaires’ references: ABCRC [62]; EORTC QLQ-C30 [51,52] M; EORTC QLQ-CR38 [24]; EORTC QLQ-CR29 ver. A [53,54]; EORTC QLQ-CR29 ver. B [55]; EORTC QLQ-ELD14 [56]; FACT-C [24]; mCOH-QOL-O ver. A [25]; mCOH-QOL-O ver. B [57,58]; QLACS [59,60,61]; QWLQ-CS [23]; SF-36v2 [21,58]; SF-12v2 [57]; WHOQOL-BREF [22]. X marks the questionnaire addresses the (sub)domain.
